# Receipt of Out-of-State Telemedicine Visits Among Medicare Beneficiaries During the COVID-19 Pandemic

**DOI:** 10.1001/jamahealthforum.2022.3013

**Published:** 2022-09-16

**Authors:** Ateev Mehrotra, Haiden A. Huskamp, Alok Nimgaonkar, Krisda H. Chaiyachati, Eric Bressman, Barak Richman

**Affiliations:** 1 Harvard Medical School, Boston, Massachusetts; 2 Beth Israel Deaconess Medical Center, Boston, Massachusetts; 3 Tufts University School of Medicine, Boston, Massachusetts; 4 University of Pennsylvania Perelman School of Medicine, Philadelphia; 5 Duke University School of Law, Durham, North Carolina

## Abstract

**Importance:**

Early in the COVID-19 pandemic, states implemented temporary changes allowing physicians without a license in their state to provide care to their residents. There is an ongoing debate at both the federal and state levels on whether to change licensure rules permanently to facilitate out-of-state telemedicine use.

**Objective:**

To describe out-of-state telemedicine use during the pandemic.

**Design, Setting, and Participants:**

This cross-sectional study of telemedicine visits included all patients with traditional Medicare from January through June 2021.

**Main Outcomes and Measures:**

Telemedicine visits from January through June 2021 where the patient’s home address and the physician’s practice address were in different states.

**Results:**

In describing which patients and specialties were using out-of-state telemedicine, we focused on the period between January to June 2021. We chose this period because it was after the turmoil of the early pandemic, when vaccines became widely available and the health care system had stabilized, but before many of the temporary licensing regulations began to lapse by mid-2021. In the first half of 2021, there were 8 392 092 patients with a telemedicine visit and, of these, 422 547 (5.0%) had 1 or more out-of-state telemedicine visits. Those who lived in a county close to a state border (within 15 miles) accounted for 57.2% of all out-of-state telemedicine visits. Among the out-of-state visits in this time period, 64.3% were with a primary care or mental health clinician. For 62.6% of all out-of-state visits, a prior in-person visit occurred between the same patient and clinician between March 2019 and the visit. The demographics and conditions treated were similar for within-state and out-of-state telemedicine visits, with several notable exceptions. Among those with a telemedicine visit, people in rural communities were more likely to receive out-of-state telemedicine care (33.8% vs 21.0%), and there was high of out-of-state telemedicine use for cancer care (9.8% of all telemedicine visits for cancer care).

**Conclusions and Relevance:**

The findings of this cross-sectional study suggest that licensure restrictions of out-of-state telemedicine would have had the largest effect on patients who lived near a state border, those in rural locales, and those who received primary care or mental health treatment.

## Key Points

**Question:**

How was out-of-state telemedicine used during the COVID-19 pandemic?

**Findings:**

In this cross-sectional study of telemedicine visits in the first half of 2021 among patients with Medicare, 422 547 patients had an out-of-state telemedicine visit; these visits were most common among those who lived near a state border and were largely for primary care and mental health treatment. In 62.6% of all out-of-state visits, a prior in-person visit occurred between the same patient and clinician.

**Meaning:**

Limitations on out-of-state telemedicine care may disrupt many existing patient-clinician relationships in primary care and mental health treatment.

## Teaser

This cross-sectional study examines out-of-state telemedicine use during the COVID-19 pandemic among Medicare beneficiaries.

## Introduction

The use of telemedicine across state lines has historically been limited, in part because of state-based licensure laws.^1,2^ Before the COVID-19 pandemic, most physicians could only care for a patient physically located in another state if the physician was also licensed in that state. Early in the pandemic, in an effort to ensure access to care, almost all states implemented temporary changes to make it easier for out-of-state physicians without a license in their state to provide care to their state’s residents.^3^

There is ongoing debate at both the federal and state levels on whether to change licensure rules permanently to facilitate out-of-state telemedicine use.^4,5,6,7^ Proposals include a national telemedicine license, allowing automatic reciprocity for licenses in other states in the Medicare program, or encouraging all states to enter the Interstate Medical Licensure Compact (IMLC). The IMLC existed before the pandemic and is an agreement among 30 states and the District of Columbia to expedite out-of-state medical licensure.^6^ Florida and Arizona have passed laws permanently making it easier for out-of-state physicians to provide telemedicine to their residents, and other states are considering reforms to licensure laws to facilitate out-of-state telemedicine.^8,9^

To inform this policy debate, we describe use of out-of-state telemedicine in the Medicare program during a time when almost all states temporarily allowed reciprocity of licensure for physicians. We describe which patients, types of clinicians, and regions of the country were most likely to use out-of-state telemedicine.

## Methods

### Identification of Telemedicine Visits

This analysis used 100% Medicare fee-for-service (FFS) claims from January 2018 through June 2021. In describing which patients and specialties were using out-of-state telemedicine, we focused on the period between January to June 2021. We chose this period because it was after the turmoil of the early pandemic, when vaccines became widely available and the health care system had stabilized, but before many of the temporary licensing regulations began to lapse by mid-2021. When these lapsed, prepandemic state-based licensing laws were effectively reinstated. As a point of comparison, we also show results from the second half of 2020 and the patterns were largely similar (eTables 7-9 in the Supplement).

The Harvard Medical School institutional review board judged this study exempt and waived informed consent because all data were deidentified. We followed the Strengthening the Reporting of Observational Studies in Epidemiology (STROBE) reporting guidelines for cross-sectional studies; although, in this descriptive study of the full Medicare sample size, we did not prespecify a sample size and do not report on missing data for all variables.^10^

We identified telemedicine visits as evaluation and management visits (visits limited to those services eligible for Medicare telemedicine reimbursement) in any setting (eg, emergency department, inpatient, office, skilled nursing facility) with any of the following telemedicine-specific codes: place of service code (02), modifier codes (GT, GQ, 95, GO), or telemedicine-specific CPT codes (G2025, 99441-3, 98966–98968, G0425-7, G0406-8, G0459, G0508-9, 0188T, G2012, G2010, 99421-99423, G2061-3, G0071, 99453, 99454, 99457, 99091, 95250-1).

### Flagging Addresses That May Not Reflect Where a Patient Is Located

A patient’s location was based on the address in the Medicare enrollment file. This address can be incorrect for several reasons. Patients with more than 1 home (eg, “snowbirders”) may live at one address and have their mail go to another, some patients may direct their mail to a child or other family member, and the address may simply be incorrect. To flag addresses that may not reflect where the patient is physically located, we first identified all in-person primary care visits. If the majority of in-person visits were with a primary care clinician (internal medicine, general practice, family practice) located more than 180 miles away, we assigned the patient the zip code of this primary care clinician. In 2021, 2.9% of patients with a telemedicine visit were assigned a new zip code, and for 79.7% the new zip code was in a different state. Among those where there was a change of state, 68.2% of these changes involved a patient switching to or away from just 3 states with a substantial number of retirees (Florida, California, Arizona). We conducted a sensitivity analysis where we excluded these beneficiaries instead of changing their designated address (eTable 5 in the Supplement).

### Patient Characteristics

Among all people receiving a telemedicine visit, we captured their age, sex, race and ethnicity, whether they resided in a rural community, original reason for Medicare eligibility (age, disability, end-stage kidney disease), and whether they were dually enrolled in Medicaid (as a marker of low-income). Race and ethnicity are self-reported to Medicare, and we used the standard Medicare categorization. We used Medicare’s definition of rural for the purposes of telemedicine, which is zip codes outside of a metropolitan Core Based Statistical Area or those in an area assigned a rural-urban commuting area code 4-10 (micropolitan to rural).^11^

### Visit Characteristics

We defined an out-of-state telemedicine visit as one where the patient’s state of residence and the clinician’s state of practice (as captured in the provider of services file) were different.

We classified telemedicine visits based on the first diagnosis code listed on the claim. Although the first diagnosis listed does not always represent the primary problem addressed in a visit, on average, it helps to characterize common conditions treated by telemedicine. We grouped visits based on the overall category (based on the first letter of the diagnosis) and the Agency for Healthcare Research and Quality Clinical Classifications Software system.^12^ For each visit, we also captured the clinician's training for the visit. We grouped clinician training into the following groups: primary care (internal medicine, family practice, general practice, geriatrics, nurse practitioners who did not specialize in mental illness), mental health specialist (psychiatrist, neuropsychiatrist, clinical psychologist, licensed clinical social worker, behavioral health nurse practitioner [identified using this methodology^13^]), uncommon specialists (hematology/oncology, rheumatology, urology, interventional cardiology, medical oncology, orthopedic surgery, neurosurgery, radiation oncology, allergy, dermatology, otolaryngology, ophthalmology, cardiac surgery, colorectal surgery, surgical oncology, maxillofacial surgery, plastic/reconstructive surgery, nuclear medicine, hand surgery), and common specialists (any physician specialist who was not in one of the other categories above). We separated mental health specialists given these clinicians have used telemedicine at a much higher rate than other specialties during the pandemic and there is policy interest in mental health specialty-specific licensure laws. We divided the remaining specialists into uncommon and common, because we hypothesized that the patterns of out-of-state telemedicine use would be higher for uncommon specialists in communities with less access to specialty care such as more rural communities.

There is ongoing debate on whether clinicians should have at least 1 in-person visit before a telemedicine visit and there have been recent legislative proposals to remove this requirement for mental health services.^14^ To determine whether a telemedicine visit during the first half of 2021 represented an initial or follow-up visit, we assessed whether there had been an in-person visit with the same clinician between January 2019 and each telemedicine visit. For example, if a patient saw a given psychiatrist for a telemedicine visit in March of 2021, we determined whether that same patient had an in-person visit with the same psychiatrist between March 2019 and that visit.

### County Characteristics

We explored the relationship between the rate of out-of-state telemedicine use in the county and the distance from the county to the closest state border and the total physicians per capita. We hypothesized that there would be greater out-of-state telemedicine use in counties close to a state border (given patients in those counties would be more likely to have existing relationships with clinicians across the border) and in counties with fewer physicians per capita (patients would be more likely to go elsewhere for care).

For each county, we calculated the percentage of telemedicine visits received by patients in that county with an out-of-state clinician. We excluded counties with fewer than 100 Medicare beneficiaries to maintain stable estimates.

### Comparison With In-Person Visits

Our primary analyses focused on telemedicine visits. As a point of comparison, we also calculated what fraction of in-person visits were with an out-of-state clinician and how this varied by the distance of the county of residence to the state border.

### Statistical Analyses

Given that we analyzed the full universe of telemedicine visits in Medicare during the study period and the large size of our study sample, we did not use statistical testing to compare differences. We did report standardized differences between patient populations to characterize the relative magnitude of the differences and categorized differences less than 0.20 as small.^15,16^All data were analyzed using SAS statistical software (version 7.5; SAS Institute, Inc).

## Results

**Figure 1.**
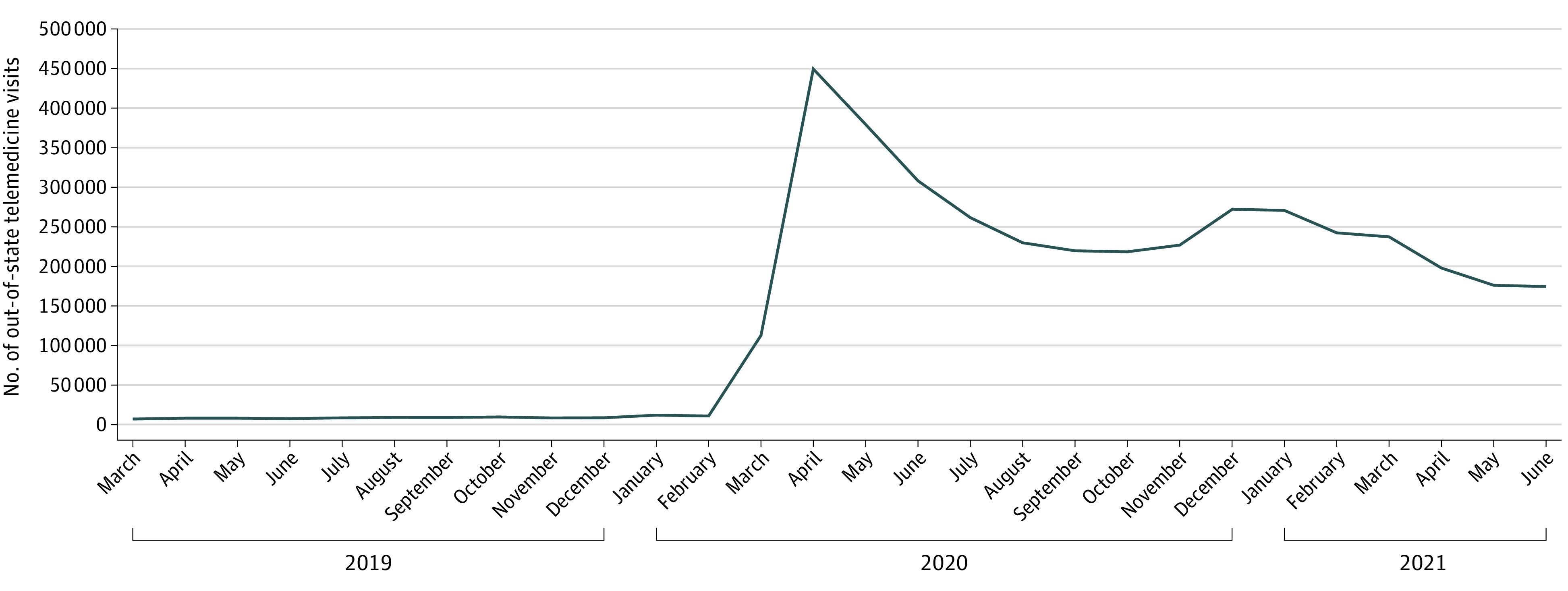
Number of Out-of-State Telemedicine Visits per Month, March 2019 to June 2021

Among Medicare beneficiaries, the number of out-of-state telemedicine visits peaked early in the pandemic (451 086, April 2020) and then slowly fell (175 545, June 2021) (Figure 1). The trend in out-of-state telemedicine visits per month was similar to the trend in total telemedicine visits (eFigure in the Supplement). The fraction of out-of-state visits was 4.5% in April 2020 and increased to 5.6% by June 2021.

In the first half of 2021, there were 8 392 092 patients with a telemedicine visit and of these, 422 547 (5.0%) had 1 or more out-of-state telemedicine visits. Compared with those who had only within-state telemedicine visits, those with out-of-state telemedicine visits were less likely to have Medicaid-dual enrollment (17.3% vs 28.0%), and more likely to live in a rural community (33.9% vs 21.1%) (standardized differences greater than 0.2) (Table 1).

**Table 1.** Characteristics of Patients With and Without Out-of-State Telemedicine Visits, January to June 2021

Characteristic	Telemedicine visits, No. (%)		Standardized difference
	Only within state	1 or more out-of-state	
No.	7 969 545	422 547	
Age, y			
<65	1 480 170 (18.6)	63 958 (15.1)	.09
65-74	3 339 523 (41.9)	188 376 (44.6)	.05
75-84	2 177 702 (27.3)	123 345 (29.2)	.04
85 or more	972 150 (12.2)	46 868 (11.1)	.03
Sex			
Male	3 225 122 (40.5)	184 378 (43.6)	.06
Female	4 744 422 (59.5)	238 169 (56.4)	.06
Race/ethnicity			
Black Non-Hispanic	725 582 (9.1)	35 170 (8.3)	.03
Hispanic	598 192 (7.5)	14 235 (3.4)	.18
White Non-Hispanic	6 050 319 (75.9)	349 595 (82.7)	.19
Other or unknown^a^	595 452 (7.5)	23 547 (5.6)	.11
Medicare eligibility			
Age	5 567 352 (69.9)	312 684 (74.0)	.09
Disability or end-stage kidney disease	2 402 193 (30.1)	109 863 (26.0)	.10
Dually insured with Medicaid			.26
No	5 738 258 (72.0)	349 631 (82.7)	
Yes	2 231 287 (28.0)	72 916 (17.3)	
Lives in a rural community			.29
No	6 288 089 (78.9)	279 528 (66.2)	
Yes	1 681 456 (21.1)	143 019 (33.9)	

^a^ Other or unknown includes Asian, North American Native, Other, and Unknown categories.

### Variation in Out-of-State Telemedicine by Geography

**Figure 2.**
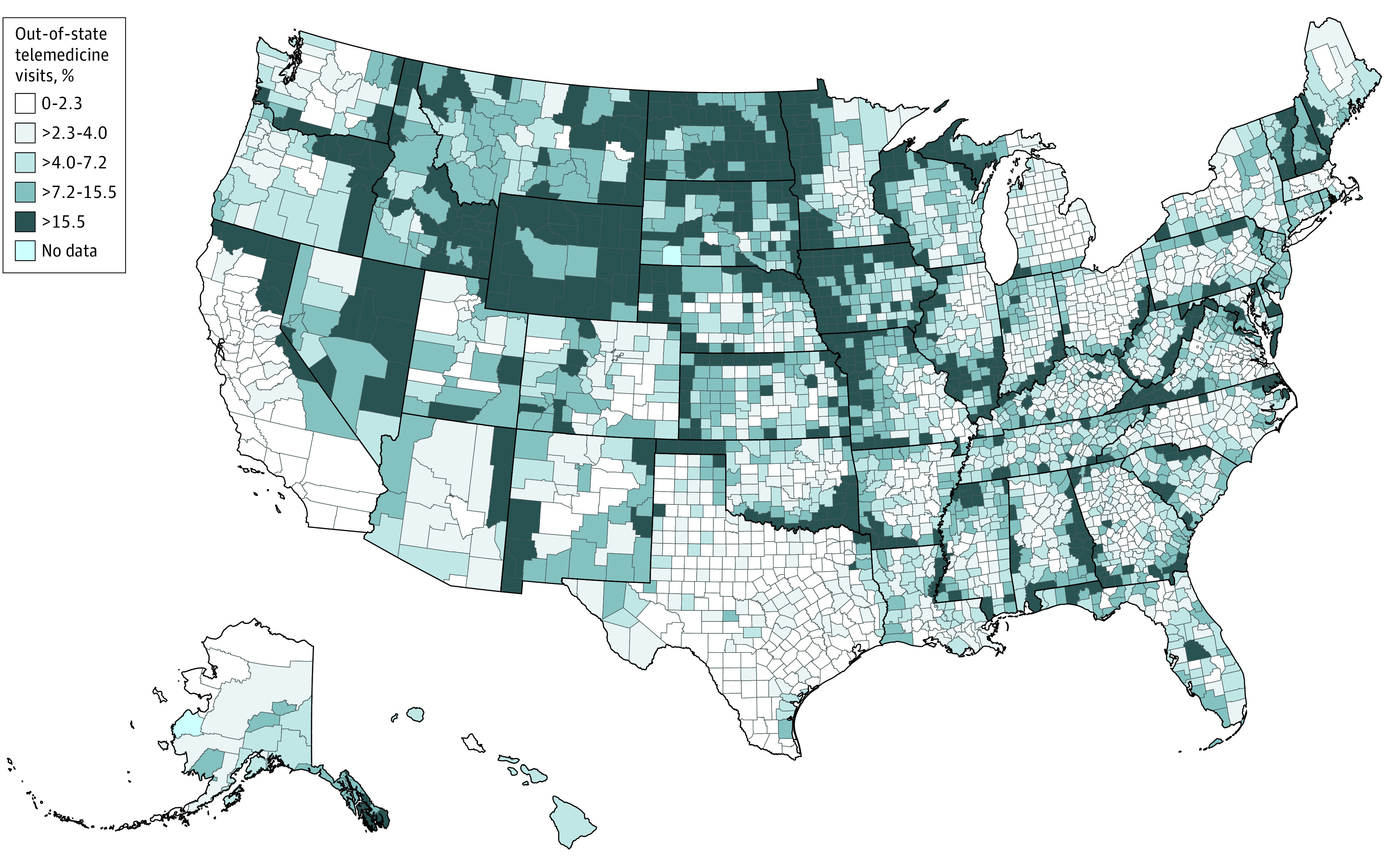
Fraction of Telemedicine Visits That Are Out of State, by County, January to June 2021

Of all Medicare beneficiaries with a telemedicine visit in January to June 2021, 33.1% lived within 15 miles of a state border and they accounted for 57.2% of all out-of-state telemedicine visits (Table 2). Counties along state borders were more likely to be in the highest quintile of counties in terms of telemedicine visits with an out-of-state clinician (Figure 2). Counties in the highest quintile of physicians per capita had fewer out-of-state telemedicine visits than counties in lower quintiles (eTable 1 in the Supplement). Among states, patients in the District of Columbia (38.5%), Wyoming (25.6%), and North Dakota (21.1%) had the highest rates of out-of-state telemedicine visits, whereas California (1.0%), Texas (2.0%), and Massachusetts (2.1%) had the lowest rates (eTable 6 in the Supplement). Typically, these visits were with a clinician in a neighboring state. For example, among patients in the District of Columbia, 92.1% of these out-of-state telemedicine visits were with clinicians in either Maryland or Virginia.

**Table 2.** Fraction of Out-of-State Telemedicine Visits, by Distance From Nearest State Border to Patient County of Residence, January to June 2021

Distance from county centroid to nearest state border, miles	All telemedicine visits, No.	Within-state telemedicine visits, No.	Out-of-state telemedicine visits, No.	Telemedicine visits in this distance category^a^ that are within state, %	Telemedicine visits in this distance category^a^ that are out-of-state, %	Within-state telemedicine visits that are in this distance category,^a^ %	Out-of-state telemedicine visits in this distance category,^a^ %	Out-of-state telemedicine visits per capita per year in this distance category,^a^ No.
0-5 Miles	2 009 261	1 780 018	229 243	88.6	11.4	10.5	22.0	0.71
6 to 10	2 185 405	1 969 705	215 700	90.1	9.9	11.7	20.7	0.59
11 to 15	1 989 930	1 838 530	151 400	92.4	7.6	10.9	14.5	0.44
16 to 20	1 576 175	1 503 871	72 304	95.4	4.6	8.9	6.9	0.29
21 to 60	5 731 106	5 504 378	226 728	96.0	4.0	32.6	21.7	0.22
61 to 120	2 690 571	2 606 886	83 685	96.9	3.1	15.4	8.0	0.16
121 to 180	637 836	615 504	22 332	96.5	3.5	3.6	2.1	0.18
>180	1 119 045	1 077 452	41 593	96.3	3.7	6.4	4.0	0.21
Total^b^	17 939 329	16 896 344	1 042 985	94.2	5.8	100	100	

^a^ We measured the distance between the centroid of the county in which the patient resided and the nearest state border. Visits were divided into categories of 0 to 5 miles up to more than 180 miles.

^b^ Total may be smaller because totals do not include visits for patients in zip codes that were not able to be categorized into county and/or distance to state border.

### Out-of-State Telemedicine by Condition and Clinician Specialty

The dominant conditions treated by out-of-state telemedicine visits during the first half of 2021 were similar to the dominant conditions treated by within-state telemedicine (38.6% vs 43.4% for the treatment of mental illness and cardiovascular disease, respectively) (Table 3). The training of clinicians providing out-of-state telemedicine vs within-state telemedicine was also similar: primary care (38.9% vs 39.3%), mental health specialists (25.4% vs 25.6%).

**Table 3.** Fraction of Out-of-State Telemedicine Visits, by Diagnosis Category, January to June 2021

Diagnosis category^a^	All telemedicine visits, No.	Within state telemedicine visits, No.	Out-of-state telemedicine visits, No.	Telemedicine visits in this distance category^b^ that are within state, %	Out-of-state telemedicine visits in this distance category,^b^ %	Within-state telemedicine visits that are in this distance category,^b^ %	Out-of-state telemedicine visits in this distance category,^b^ %
Cancer	693 019	624 966	68 053	90.2	9.8	2.7	5.2
Hematology	353 046	330 250	22 796	93.5	6.5	1.4	1.8
Neurological disease	1 546 796	1 451 319	95 477	93.8	6.2	6.3	7.4
Gastrointestinal	659 699	622 313	37 386	94.3	5.7	2.7	2.9
Musculoskeletal	2 323 511	2 196 253	127 258	94.5	5.5	9.5	9.8
Cardiovascular disease	3 099 965	2 934 131	165 834	94.7	5.3	12.7	12.8
Diabetes and other endocrine	2 226 200	2 110 947	115 253	94.8	5.2	9.2	8.9
Genitourinary	830 067	789 308	40 759	95.1	4.9	3.4	3.1
Pulmonary disease	908 105	866 326	41 779	95.4	4.6	3.8	3.2
Mental illness	7 409 180	7 074 572	334 608	95.5	4.5	30.7	25.8
Other	4 286 064	4 036 638	249 426	94.2	5.8	17.5	19.2
Total	24 335 652	23 037 023	1 298 629	94.7	5.3	100	100

^a^ Diagnosis category designated by first letter of *International Statistical Classification of Diseases and Related Health Problems, Tenth Revision* diagnosis code on visit.

^b^ We measured the distance between the centroid of the county in which the patient resided and the nearest state border. Visits were divided into categories of 0 to 5 miles up to more than 180 miles.

However, there were some notable exceptions. Use of out-of-state telemedicine was higher for some specialized care. The rate of out-of-state telemedicine use was highest for cancer care (9.8%) (Table 3). When we use more granular diagnosis groupings, the top 3 conditions in terms of rates of out-of-state telemedicine use were: assessment of organ transplant (13.0%), male reproductive cancers such as prostate cancer (11.3%), and graft-related issues (10.2%) (eTable 2 in the Supplement). The rates of out-of-state telemedicine were highest for specialized care: uncommon specialists (8.5%), common specialists (6.4%), mental health specialists (4.4%), and primary care (4.2%) (rates broken down by individual specialty in eTable 3 in the Supplement).

### Receipt of a Prior In-Person Visit

Across all out-of-state telemedicine visits in the first half of 2021, we observed a prior in-person visit between March 2019 and the visit with the same patient and same clinician in 62.8.% of those visits. In contrast, across all within-state telemedicine visits, we observed a prior in-person visit in 75.8% of visits.

### Comparison to In-Person Visits

The rate of out-of-state in-person visits was similar to the rate of out-of-state telemedicine visits. For example, among those who lived within 5 miles of a state border, the rate of out-of-state in-person visits was 10.5% vs 11.4% of telemedicine visits (eTable 4 in the Supplement).

## Discussion

There have been limited data to inform the ongoing debate on permanently changing physician licensure laws to facilitate out-of-state telemedicine. We conducted a cross-sectional study of telemedicine visits during a period of the pandemic when there were fewer state licensure limitations on out-of-state telemedicine. Among all traditional Medicare beneficiaries with a telemedicine visit in the first half of 2021, 5.0% received an out-of-state telemedicine visit. The majority of out-of-state telemedicine visits were among patients who live in a county within 15 miles of a state border. In general, patient demographics, conditions treated, and clinician specialty were similar between within-state and out-of-state telemedicine visits. Also, for both out-of-state and within-state telemedicine visits, in most cases, we observed a prior in-person visit between the same patient and clinician. There were several notable exceptions. We observed greater use of out-of-state telemedicine care in rural communities and for some less common conditions such as cancer.

The results of this cross-sectional study echo and advance prior research on the use of telemedicine during the pandemic. The initial spike and then gradual decline in out-of-state telemedicine visits we observed in the pandemic largely follow the trends observed in the pandemic across all telemedicine visits in Medicare,^17^ commercial health plans,^18,19^ and other data sources.^20^ In these analyses we find that a large fraction of out-of-state telemedicine visits were for mental illness. Prior work has found that, in general, telemedicine use has been higher for the treatment of mental illness and that mental health specialists have embraced telemedicine at higher rates than other clinical specialties.^17,18,20^ A recent study by Andino and colleagues on out-of-state telemedicine also finds an initial spike and then decline, with roughly 5% of all telehealth visits being out-of-state, and that those with out-of-state telemedicine are more likely to live in rural communities and less likely to be dually insured in Medicaid.^21^ Our research extends that research by describing trends into 2021, emphasizing that the greatest telemedicine use was among those close to a state border, and describing the variation of out-of-state telemedicine by specialty of the clinician providing the visit.

These results highlight which populations might be differentially affected by restrictions on out-of-state telemedicine. Relaxation of state restrictions would likely offer immediate convenience to patients who live near a state border and those receiving primary care and mental health treatment. These patients are subject to an accident of geography; 2 patients receiving the same care may have very different experiences. A patient with a primary care physician who lives in the middle of a state can access care via telemedicine. However, a similar patient living near a state border with a primary care physician in the neighboring state now will have to physically travel to that appointment. This is best illustrated among those who live in the District of Columbia, which had the highest rate of out-of-state telemedicine visits (38.5%) with more than 90% of these out-of-state telemedicine visits with clinicians in neighboring Maryland or Virginia.

A second population more likely to capitalize on a relaxation of state telemedicine restrictions are rural beneficiaries and those who live in counties with fewer physicians per capita. We believe these differences are partly driven by the lack of availability of specialty clinicians in the local community in rural areas and specifically the lack of availability of tertiary care centers.^22,23,24^ This likely explains why we saw greater use of out-of-state telemedicine for conditions such as cancer and conditions treated by uncommon specialists. As state licensure regulations change, it will be important to track telemedicine use among these populations to assess if use differentially falls.

There have been many calls as well as proposals to change licensure laws to facilitate out-of-state telemedicine.^25^ To the degree that the patterns of out-of-state telemedicine we observed in 2021 are a prologue to the future, they imply that regional compacts would have a similar effect as more sweeping reforms such as a national license. They also might be politically more feasible. In a regional compact, a given state would create a reciprocity compact with its neighboring state in which a physician licensed in either state would have automatic licensure reciprocity in the other state. Such a local compact was recently proposed for Maryland, Virginia, and the District of Columbia.^26^

### Limitations

There are many limitations of our analyses. First, it is limited to the traditional Medicare population. It is possible that among a younger population with commercial insurance, both in-state and out-of-state telemedicine may be higher. Second, we determined whether a visit was out-of-state based on the patient’s home address and the clinician’s practice address. Although we addressed some discrepancies in these data, both could still have inaccuracies. For example, the clinician’s address could be where billing and payment are processed (eg, the main hub of a health system or a telemedicine services company) and not where clinical care is provided. Also, we assumed that the patient was located at their home for the telemedicine visit, but patients may physically be located in another state at the time of the visit (for example, on vacation). All states (except possibly Ohio) implemented changes to make it easier for out-of-state physicians to provide care within the state, but the changes themselves were quite heterogeneous, ranging from automatic reciprocity to issuing temporary telehealth-only licenses.^3^ We do not account for these differences when comparing rates of out-of-state telemedicine use. Finally, we focused on patients with within-state and out-of-state telemedicine visits and therefore did not account for differences in which patients have any telemedicine visits.

## Conclusions

There are ongoing debates about whether to permanently change licensure regulations to facilitate out-of-state telemedicine visits. We find that during a period where licensure regulations were temporarily waived, out-of-state telemedicine visits were common and used most by patients who live near state borders or in rural communities, those receiving primary care services and mental health treatment, and those receiving cancer care.

## Notes

Supplement. **eTable 1.** Rates of out-of-state telemedicine across counties stratified by number of physicians per capita in county, January-June 2021 **eTable 2.** Top and bottom ten conditions in terms of rates of out-of-state telemedicine use, January–June 2021 **eTable 3.** Rates of out-of-state telemedicine by specialty/training (limited to top 20), January–June 2021 **eTable 4.** Fraction of in-person visits that are with an out-of-state physician, January–June 2021 **eTable 5.** Comparison of the fraction of in-person visits that are with an out-of-state clinician under two scenarios, January – June 2021 **eTable 6.** Fraction of telemedicine visits that are out-of-state, by state, January – June 2021 **eTable 7.** Comparison of characteristics of patients with and without out-of-state telemedicine visits in January – June 2021 and July – December 2020 **eTable 8.** Comparison of fraction of telemedicine visits that are out-of-state, by distance from nearest state border to patient’s county of residence, January-June 2021 and July – December 2020 **eTable 9.** Fraction of telemedicine visits that are out-of-state, by diagnosis category, January – June 2021 and July – December 2020 **eFigure 1.** Total (in-state and out-of-state) telemedicine visits per month and fraction that are out of state
