# Errors in Header Text in Table 3

**DOI:** 10.1001/jamahealthforum.2022.4353

**Published:** 2022-11-04

In the article titled “Receipt of Out-of-State Telemedicine Visits Among Medicare Beneficiaries During the COVID-19 Pandemic”^1^ published on September 16, 2022, in *JAMA Health Forum*, there were errors in some of the header text in Table 3. In 4 places where it previously read “distance category” it now correctly reads “diagnosis category.” This article was corrected online.
