# Supplementary material for: Receipt of Out-of-State Telemedicine Visits Among Medicare Beneficiaries During the COVID-19 Pandemic
Source: JAMA Health Forum. 2022 Sep 16;3(9):e223013. doi: 10.1001/jamahealthforum.2022.3013 (PMC9482058; doi:10.1001/jamahealthforum.2022.3013)
Supplement: Supplement. — eTable 1. Rates of out-of-state telemedicine across counties stratified by number of physicians per capita in county, January-June 2021 eTable 2. Top and bottom ten conditions in terms of rates of out-of-state telemedicine use, January–June 2021 eTable 3. Rates of out-of-state telemedicine by specialty/training (limited to top 20), January–June 2021 eTable 4. Fraction of in-person visits that are with an out-of-state physician, January–June 2021 eTable 5. Comparison of the fraction of in-person visits that are with an out-of-state clinician under two scenarios, January – June 2021 eTable 6. Fraction of telemedicine visits that are out-of-state, by state, January – June 2021 eTable 7. Comparison of characteristics of patients with and without out-of-state telemedicine visits in January – June 2021 and July – December 2020 eTable 8. Comparison of fraction of telemedicine visits that are out-of-state, by distance from nearest state border to patient’s county of residence, January-June 2021 and July – December 2020 eTable 9. Fraction of telemedicine visits that are out-of-state, by diagnosis category, January – June 2021 and July – December 2020 eFigure 1. Total (in-state and out-of-state) telemedicine visits per month and fraction that are out of state [file jamahealthforum-e223013-s001.pdf]

Supplemental Online Content

Mehrotra A, Huskamp HA, Nimgaonkar A, et al. Receipt of out-of-state telemedicine visits among Medicare beneficiaries during the COVID-19 pandemic. *JAMA Health Forum*. 2022;3(9):e223013. doi:10.1001/jamahealthforum.2022.3013

eTable 1. Rates of out-of-state telemedicine across counties stratified by number of physicians per capita in county, January-June 2021
eTable 2. Top and bottom ten conditions in terms of rates of out-of-state telemedicine use, January–June 2021
eTable 3. Rates of out-of-state telemedicine by specialty/training (limited to top 20), January–June 2021
eTable 4. Fraction of in-person visits that are with an out-of-state physician, January–June 2021
eTable 5. Comparison of the fraction of in-person visits that are with an out-of-state clinician under two scenarios, January – June 2021
eTable 6. Fraction of telemedicine visits that are out-of-state, by state, January – June 2021
eTable 7. Comparison of characteristics of patients with and without out-of-state telemedicine visits in January – June 2021 and July – December 2020
eTable 8. Comparison of fraction of telemedicine visits that are out-of-state, by distance from nearest state border to patient's county of residence, January-June 2021 and July – December 2020
eTable 9. Fraction of telemedicine visits that are out-of-state, by diagnosis category, January – June 2021 and July – December 2020
eFigure 1. Total (in-state and out-of-state) telemedicine visits per month and fraction that are out of state

This supplemental material has been provided by the authors to give readers additional information about their work.

© 2022 Mehrotra A, et al. *JAMA Health Forum.*

eTable 1. Rates of out-of-state telemedicine across counties stratified by number of physicians per capita in county, January – June 2021

Physicians per capita (Quintile of counties)	Total telemedicine visits	Percentage of Telemedicine Visits that are out-of-state
Lowest MD per capita (<0.3 MD per 1000)	504,396	8.1%
2nd	913,208	9.0%
3rd	1,653,531	7.8%
4th	4,440,180	7.1%
Highest MD per capita (>1.8 MD per 1000)	16,395,892	4.3%

© 2022 Mehrotra A, et al. *JAMA Health Forum.*

eTable 2. Top and bottom ten conditions in terms of rates of out-of-state telemedicine use, January – June 2021

Top 10	Total Visits	Out-of-State Visits	%
Organ transplant status	44,422	5,797	13.0%
Male reproductive system cancers - prostate	120,476	13,621	11.3%
Implant, device or graft related encounter	40,607	4,127	10.2%
Personal/family history of disease	111,565	10,420	9.3%
Cerebral infarction	58,390	5,429	9.3%
Parkinson`s disease	108,022	9,351	8.7%
Other nervous system disorders (often hereditary)	52,258	4,393	8.4%
Respiratory failure; insufficiency; arrest	45,835	3,811	8.3%
Nonrheumatic and unspecified valve disorders	57,773	4,691	8.1%
Benign neoplasms	50,884	4,116	8.1%
**Bottom 10**			
Nutritional anemia	69,481	2,804	4.0%
Hypertension with complications and secondary	69,354	2,764	4.0%
Viral infection	41,786	1,651	4.0%
Neurodevelopmental disorders	201,102	7,924	3.9%
Nutritional deficiencies	50,726	1,930	3.8%
Sinusitis	122,832	4,377	3.6%
Schizophrenia spectrum and other psychotic dis	774,780	24,012	3.1%
Other specified upper respiratory infections	87,225	2,632	3.0%
Disruptive, impulse-control and conduct disorder	46,044	1,373	3.0%
Acute bronchitis	47,070	1,379	2.9%

© 2022 Mehrotra A, et al. *JAMA Health Forum.*

eTable 3: Rates of out-of-state telemedicine by specialty/training (limited to top 20), January – June 2021

Specialty of Provider	Total telemedicine visits	Percentage of Telemedicine Visits that are out-of-state	Percentage of all out-of-state Telemedicine Visits	Percentage of all within state telemedicine visits
Urology	265,613	8.8%	1.8%	1.1%
Neurology	626,563	7.9%	3.8%	2.5%
Phys Med/Rehab	270,324	7.4%	1.5%	1.1%
Hematology/oncology	325,297	7.2%	1.8%	1.3%
Rheumatology	279,700	7.1%	1.5%	1.1%
Interventional Cardiology	162,670	7.1%	0.9%	0.7%
Physical therapist	166,707	6.8%	0.9%	0.7%
Gastroenterology	488,704	6.6%	2.5%	2.0%
Endocrinology	587,480	6.3%	2.9%	2.4%
Nurse practitioner	3,347,996	6.3%	16.2%	13.8%
Sleep Medicine	143,267	6.0%	0.7%	0.6%
Pulmonary disease	396,422	5.8%	1.8%	1.6%
Clin Psych	1,220,956	5.5%	5.2%	5.1%
Nephrology	502,452	5.4%	2.1%	2.1%
Phys. Asst.	976,233	5.3%	4.0%	4.1%
Interventional Pain Management	179,593	5.2%	0.7%	0.7%
Pain mgmt	282,922	4.6%	1.0%	1.2%
Psychiatry	2,363,529	4.4%	8.1%	9.9%
Licensed clinical social worker	1,845,319	4.2%	6.0%	7.7%
Primary care	6,776,933	3.5%	18.1%	28.7%

© 2022 Mehrotra A, et al. *JAMA Health Forum.*

eTable 4. Fraction of *in-person visits* that are with an out-of-state physician, January – June 2021

Distance from county to nearest state border	Total in-person visits	Fraction of visits that are out-of-state	Fraction of all out-of-state in-person visits
0 to 5 Miles	21,627,083	10.5%	19.3%
6 to 10	25,329,894	9.8%	20.9%
11 to 15	24,332,713	7.5%	15.5%
16 to 20	16,465,483	5.4%	7.5%
21 to 60	71,871,871	3.6%	22.1%
61 to 120	34,804,343	2.8%	8.2%
121 to 180	9,033,676	3.1%	2.4%
> 180	13,564,171	3.6%	4.1%

© 2022 Mehrotra A, et al. *JAMA Health Forum.*

eTable 5. Comparison of the fraction of in-person visits that are with an out-of-state clinician under two scenarios, January – June 2021

As described in methods, a small fraction of Medicare beneficiaries appeared to have mistaken addresses. In our primary analyses in the manuscript (scenario 1), we changed their address to the zip code of their primary care physician. In a sensitivity analysis (scenario 2), we excluded these beneficiaries.

The results were largely similar.

**Month in 2021**	**Address changed** (primary strategy in manuscript)	**Excluding these beneficiaries** (sensitivity analysis)
January	5.7%	5.6%
February	5.7%	5.7%
March	5.6%	5.6%
April	5.4%	5.4%
May	5.2%	5.2%
June	5.2%	5.2%

© 2022 Mehrotra A, et al. *JAMA Health Forum.*

eTable 6. Fraction of telemedicine visits that are out-of-state, by state, January – June 2021

California	1.1%
Texas	2.1%
Massachusetts	2.2%
Michigan	2.7%
Hawaii	3.1%
New York	3.2%
Ohio	3.9%
Arizona	4.0%
Colorado	4.1%
Utah	4.3%
North Carolina	4.6%
Louisiana	5.0%
Illinois	5.3%
Pennsylvania	5.5%
Minnesota	5.6%
Washington	5.8%
Oregon	6.0%
New Mexico	6.2%
Arkansas	6.3%
Kentucky	6.4%
Georgia	6.4%
Tennessee	6.4%
Florida	6.6%
Oklahoma	7.2%
Connecticut	7.4%
Indiana	7.6%
Wisconsin	7.8%
Nevada	8.1%
Puerto Rico	8.3%
New Jersey	8.6%
Alabama	8.8%
Kansas	8.9%
Montana	9.0%
Nebraska	9.0%
Alaska	9.2%
Mississippi	9.2%
Virginia	9.6%
Maine	9.8%
Maryland	9.9%
Missouri	11.0%
Rhode Island	11.2%
South Carolina	11.5%
Delaware	11.8%
Idaho	13.8%
South Dakota	14.6%
Iowa	14.9%
West Virginia	15.0%
New Hampshire	15.8%
Vermont	16.6%
Virgin Islands	16.9%
North Dakota	21.4%
Wyoming	25.6%
District of Columbia	38.5%

© 2022 Mehrotra A, et al. *JAMA Health Forum.*

eTable 7. Comparison of characteristics of patients with and without out-of-state telemedicine visits in January – June 2021 and July – December 2020

	Jan - Jun 2021		July - Dec 2020	
	Only within state telemedicine visits	One or more out-of-state telemedicine visits	Only within state telemedicine visits	One or more out-of-state telemedicine visits
	n=7,969,545	n=422,547	n=9,999,031	n=448,114
	%	%	%	%
Age				
<65	18.6	15.1	17.9	14.6
65-74	41.9	44.6	43.4	45.9
75-84	27.3	29.2	26.6	28.2
85 or more	12.2	11.1	12.1	11.2
Sex				
Male	40.5	43.6	40.8	44
Female	59.5	56.4	59.2	56
Race & Ethnicity				
White Non-Hispanic	75.9	82.7	76	83.2
Black Non-Hispanic	9.1	8.3	8.8	8.3
Hispanic	7.5	3.4	7.5	3.2
Other or unknown	7.5	5.6	7.7	5.3
Medicare eligibility				
Age	69.9	74	71.4	75
Disability or End Stage Renal Disease	30.1	26	28.6	25
Dually insured with Medicaid				
No	72	82.7	73.4	83.6
Yes	28	17.3	26.6	14.4
Live in a rural community				
No	78.9	66.2	78.2	65.6
Yes	21.1	33.9	21.8	32.4

© 2022 Mehrotra A, et al. *JAMA Health Forum.*

eTable 8. Comparison of fraction of telemedicine visits that are out-of-state, by distance from nearest state border to patient's county of residence, January-June 2021 and July – December 2020

	Jan - Jun 2021			Jul - Dec 2020		
Distance from county to nearest state border	% of telemedicine visits that are out-of-state	% of all out-of-state telemedicine visits	% of all within state telemedicine visits	% of telemedicine visits that are out-of-state	% of all out-of-state telemedicine visits	% of all within state telemedicine visits
0 to 5 Miles	11.4%	22.0%	10.5%	10.6%	22.8%	9.9%
6 to 10	9.9%	20.7%	11.7%	9.4%	22.5%	11.2%
11 to 15	7.6%	14.5%	10.9%	6.7%	15.0%	10.7%
16 to 20	4.6%	6.9%	8.9%	3.9%	6.7%	8.4%
21 to 60	4.0%	21.7%	32.6%	3.1%	20.1%	32.4%
61 to 120	3.1%	8.0%	15.4%	2.2%	6.9%	16.0%
121 to 180	3.5%	2.1%	3.6%	2.4%	1.9%	4.0%
> 180	3.7%	4.0%	6.4%	2.8%	4.1%	7.3%

© 2022 Mehrotra A, et al. *JAMA Health Forum.*

eTable 9. Fraction of telemedicine visits that are out-of-state, by diagnosis category, January – June 2021 and July – December 2020

	Jan-Jun 2021				Jul-Dec 2020			
**Diagnosis category***	**Total telemedicine visits**	**% of telemedicine visits that are out-of-state**	**% of all out-of-state telemedicine visits**	**% of all within state telemedicine visits**	**Total telemedicine visits**	**% of telemedicine visits that are out-of-state**	**% of all out-of-state telemedicine visits**	**% of all within state telemedicine visits**
Cancer	693,019	9.80%	5.24%	2.71%	854,234	9.49%	5.68%	2.63%
Hematology	353,046	6.50%	1.76%	1.43%	444,058	6.39%	1.99%	1.42%
Neurological disease	1,546,796	6.20%	7.35%	6.30%	1,927,833	5.32%	7.19%	6.22%
Gastrointestinal	659,699	5.70%	2.88%	2.70%	837,621	4.86%	2.85%	2.71%
Musculoskeletal	2,323,511	5.50%	9.80%	9.53%	3,015,953	4.75%	10.04%	9.79%
Cardiovascular disease	3,099,965	5.30%	12.77%	12.74%	4,042,512	4.63%	13.11%	13.13%
Diabetes and other endocrine	2,226,200	5.20%	8.87%	9.16%	2,804,690	3.90%	7.67%	9.18%
Genitourinary	830,067	4.90%	3.14%	3.43%	1,133,401	4.24%	3.37%	3.70%
Pulmonary disease	908,105	4.60%	3.22%	3.76%	1,227,056	4.24%	3.64%	4.00%
Mental illness	7,409,180	4.50%	25.77%	30.71%	8,324,867	3.84%	22.40%	27.27%

© 2022 Mehrotra A, et al. *JAMA Health Forum.*

eFigure 1. Total (in-state and out-of-state) telemedicine visits per month and fraction that are out of state

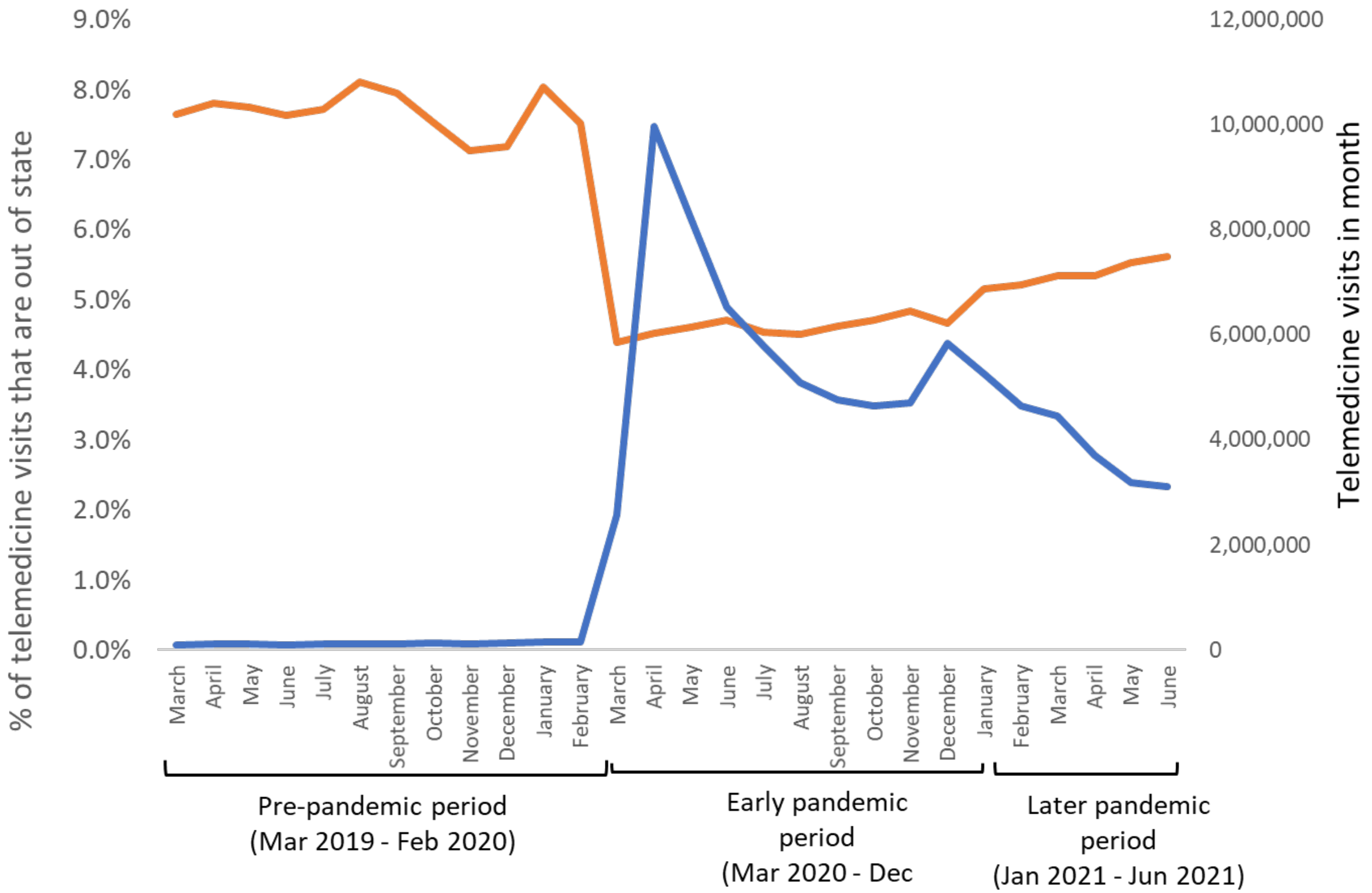

© 2022 Mehrotra A, et al. *JAMA Health Forum.*
